# Anxiety and Academic Procrastination in Deaf and Hard of Hearing College Students: A Moderated Mediation Model

**DOI:** 10.3390/bs14121219

**Published:** 2024-12-18

**Authors:** Guomin Li, Zhiheng Xiong, Pingting Lin

**Affiliations:** 1 School of Education Science, Nanjing Normal University of Special Education, Nanjing 210038, China; 225026@njts.edu.cn; 2 School of Humanities, Southeast University, Nanjing 211189, China; xzh_psy@seu.edu.cn; 3 School of Special Education, Nanjing Normal University of Special Education, Nanjing 210038, China

**Keywords:** deaf and hard of hearing, college students, psychological resilience, a moderated mediation model

## Abstract

Deaf and hard of hearing college students encounter unique challenges and pressures in their daily lives and academic pursuits, often leading to heightened anxiety levels, which may increase the likelihood of academic procrastination. This study aims to investigate the relationship between anxiety and academic procrastination in deaf and hard of hearing college students, with a focus on the mediating role of rumination and the moderating effect of psychological resilience. The findings offer valuable insights into strategies for reducing anxiety and academic procrastination in this population. A total of 685 deaf and hard of hearing college students were assessed using the Generalized Anxiety Disorder Scale, Academic Procrastination Scale, Rumination Scale, and Psychological Resilience Scale. The study revealed three key findings: (1) Anxiety is a significant positive predictor of academic procrastination in deaf and hard of hearing college students; (2) Rumination partially mediates the relationship between anxiety and academic procrastination, suggesting that anxiety indirectly influences procrastination through rumination; (3) Psychological resilience moderates the relationship between rumination and academic procrastination, with higher levels of resilience diminishing the impact of rumination on procrastination. The findings of this study provide a deeper understanding of the complex relationship between anxiety and procrastination in deaf and hard of hearing college students, within the context of ecosystem theory and resilience theory of development.

## 1. Introduction

In March 2021, the World Health Organization’s World Hearing Report estimated that 1.5 billion people globally are living with hearing loss, and by 2050, this number is projected to reach nearly 2.5 billion [1]. With the growth of inclusive education, opportunities for deaf and hard of hearing college students in China to access higher education have steadily increased. However, research from various countries indicates that while the enrollment rate of deaf and hard of hearing students has risen significantly, the proportion of those completing their degrees remains low [2]. Studies show that compared to their hearing peers, deaf and hard of hearing students often perform worse academically, progress more slowly, and face a higher risk of dropping out [1]. Hearing loss impacts their language development, cognitive growth, and social functioning, creating additional challenges, particularly for deaf and hard of hearing students in mainstream educational settings [3,4]. Deaf and hard of hearing children frequently lag behind their peers in reading ability, and they often require more time and effort to process information [5].

The root cause of these educational problems lies in the lack of language exposure in the early years of children’s lives. There is growing evidence that this lack of language exposure has a significant negative impact on children’s development [6]. For example, deaf and hard of hearing children generally show a serious delay in language development when they enter school [7]. This phenomenon not only predicts the learning challenges they may face in the future, but may also be an important social factor that triggers mental health problems [8]. When adequate support systems are not in place, these challenges can lead to further learning difficulties and emotional problems. Therefore, while expanding educational access is essential, it does not fully address the broader challenges faced by deaf and hard of hearing students in higher education. The emotional and academic difficulties they encounter throughout their studies warrant deeper attention.

Previous research has primarily focused on the direct relationship between anxiety and academic procrastination, with limited investigation into the underlying mechanisms [9,10,11]. Additionally, there is a lack of research on these processes in the deaf and hard of hearing college student population. This study introduces rumination as a factor to explore its mediating role between anxiety and academic procrastination. If anxiety influences procrastination through rumination, it is important to ask whether other moderating factors could enrich our understanding of this relationship. This study examines the crucial role of psychological resilience in this context. Psychological resilience is a key factor that enables individuals to adapt to adversity. Research shows that resilience, built on cognitive and socio-emotional skills, interacts with negative life events and can be observed through positive learning and social behaviors [12]. This study aims to further explore the relationship between anxiety and academic procrastination in deaf and hard of hearing college students, enhancing our understanding of the psychological mechanisms at play and providing a foundation for the prevention and intervention of academic procrastination in this population.

## 2. Literature Review

### 2.1. Anxiety and Academic Procrastination in Deaf and Hard of Hearing College Students

Anxiety refers to the unpleasant emotional state individuals experience in response to a series of stimuli or events, often accompanied by heightened autonomic nervous system activity [13]. While moderate anxiety serves an adaptive function as a basic human emotion, elevated levels of anxiety can significantly impair cognitive functioning [14,15]. Like their hearing peers, deaf and hard of hearing college students are in a critical stage of life, facing challenges in academics, employment, and social interactions. Research has identified several risk factors for anxiety, including cognitive and functional impairments, inadequate social support, loneliness, and traumatic experiences, all of which are linked to hearing loss [16,17]. In addition, deaf and hard of hearing students may face a chronic lack of speech [18,19]. Studies have shown that failing to speak can have a lasting effect on neurological development [20]. In particular, the development of the nervous system may be altered, making it difficult for deaf and hard of hearing students to develop sufficient language skills to support fluent communication [6]. Additionally, deaf and hard of hearing students may face difficulties with executive functioning, including understanding verbal instructions, maintaining attention, and navigating social norms. These challenges can result in missed information during the learning process, requiring more time and effort to comprehend and process material, ultimately making it harder to stay focused and impacting academic performance [21]. In summary, the ongoing academic pressures and communication challenges may undermine their confidence, leading to feelings of helplessness and frustration, which can cause psychological stress and anxiety [22]. Given their unique needs in communication and learning, deaf and hard of hearing students often require additional attention and support. When these necessary support systems are lacking, feelings of helplessness and frustration can intensify, further exacerbating psychological stress and anxiety. Academic procrastination is a prevalent issue among college students, defined as the intentional delay of starting or completing academic tasks [23]. According to attentional control theory, anxiety impairs cognitive functions such as attention control and working memory, making it difficult to focus on academic tasks or manage anxiety-related intrusive thoughts [24]. This can increase the time and effort required for learning, thereby raising the likelihood of academic procrastination. The Short-term Mood Regulation Theory suggests that procrastination is an ineffective coping strategy for anxiety, as individuals tend to prioritize managing their emotional state over completing long-term tasks with positive outcomes, such as studying [25,26]. Previous research has consistently shown a positive correlation between anxiety and procrastination, with higher levels of anxiety often leading to increased procrastination [27]. Based on this discussion, this study proposes Hypothesis 1: Anxiety among deaf and hard of hearing college students significantly predicts their procrastination behavior.

### 2.2. The Mediating Role of Rumination Between Anxiety and Academic Procrastination in Deaf and Hard of Hearing College Students

Rumination is a negative cognitive response characterized by excessive and repetitive focus on one’s negative emotions, their causes, and adverse consequences, without the motivation to resolve these issues [28]. Research indicates that while rumination affects hearing college students, deaf and hard of hearing students face additional individual challenges and systemic barriers [29,30]. Ecosystem theory suggests that various subsystems, such as family, school, and peers, play a significant role in individual development [31,32]. Mental health is shaped by cumulative and interacting risk factors in a person’s environment [33]. They are more likely to face early language deprivation, educational and communication conflicts within the family, insufficient or inappropriate parental involvement, and challenges in accessing sign language services [34]. For example, the concept of “dinner table syndrome” illustrates the communication deprivation experienced by deaf and hard of hearing individuals, particularly those born into hearing families, where they are often excluded from everyday conversations [35]. Research has shown that family dynamics significantly influence rumination in adolescents. When parents demonstrate neglect or rejection, it diminishes the child’s sense of autonomy and control over their environment, which in turn increases the likelihood of rumination [28]. A comparative study involving 326 deaf and hard of hearing students and 393 hearing students in China found that deaf and hard of hearing students reported higher levels of rumination and relied more on negative coping strategies than their hearing counterparts [36]. Rumination may therefore act as a key mediator between anxiety and procrastination behaviors. Studies have demonstrated that rumination mediates the relationship between anxiety and procrastination, with its effect being more pronounced than that of worry [37]. Both early cognitive interference and processing efficiency theories, along with more recent attentional control theory, suggest that anxiety impairs inhibitory control [15,38]. Inhibitory control, a critical function of the central executive system, refers to an individual’s ability to regulate their attention, behavior, thoughts, or emotions by suppressing strong internal tendencies or external distractions in favor of more appropriate or necessary actions [39]. Impairment in this cognitive function may prevent individuals from managing anxiety-related intrusive thoughts, thereby fostering rumination. Empirical evidence has shown a strong positive correlation between rumination and procrastination [37,40]. Thus, based on these findings, this study proposes Hypothesis 2: Rumination mediates the relationship between anxiety and academic procrastination.

### 2.3. The Moderating Role of Psychological Resilience Between Rumination and Academic Procrastination in Deaf and Hard of Hearing College Students

Psychological resilience is a fundamental concept in positive psychology, referring to an individual’s ability to adapt effectively to life challenges such as stress, frustration, and trauma [41,42]. It encompasses successful adaptation in adversity, achieving positive outcomes in high-risk situations, and maintaining resilience in the face of threats [43]. Given its significance for psychological well-being, psychological resilience has been studied across various populations. It is particularly important to explore the resilience of deaf and hard of hearing students, as hearing loss and societal attitudes place them in high-risk environments [44]. In recent years, international perspectives on “disability” have shifted from a deficit-based medical model to a social–ecological model centered on human functioning [45]. Under this framework, “disability” and “deafness” are no longer understood solely through the lens of disease and impairment [46]. Against this background, scholars have applied ecosystem theory and resilience theory of development to explore the factors associated with positive higher education outcomes for deaf and hard of hearing individuals. These theories emphasize the importance of recognizing the strengths of deaf and hard of hearing individuals, while considering the interactions and influences between various elements within their ecosystems. By ensuring the adaptability and stability of these systems in response to external environmental changes, development can be fostered through effective support services and continuous improvement of macro-level policies [32]. Ecosystem and resilience theories provide new perspectives for research. Rather than solely focusing on the outcomes of psychological resilience, we must carefully examine the factors that can protect individuals from negative influences. The protective factor model of psychological resilience suggests that resilience plays a crucial role in moderating the interaction between individuals and risk environments. By promoting the effective regulation of cognition and emotions, resilience enhances adaptability to one’s environment and can mitigate the negative impact of risk factors [47]. Studies have demonstrated that individuals with higher resilience tend to employ more positive cognitive and emotional regulation strategies, such as cognitive reappraisal, self-affirmation, and meaning-making, to cope with stress [48]. In contrast, rumination—characterized by sustained and passive cognitive processing of negative events—weakens problem-solving abilities. Psychological resilience can act as a protective factor, buffering against the negative consequences of this cognitive chain reaction [30]. Therefore, psychological resilience is likely to mitigate the adverse effects of rumination on academic procrastination, playing a pivotal role in this relationship. This study introduces psychological resilience as an intrinsic quality and, building on prior research that often considers resilience a moderating variable, examines whether the resilience resources of deaf and hard of hearing college students function as a protective factor in the relationship between rumination and academic procrastination. Moreover, this research seeks to integrate personal resources and social support systems that facilitate resilience development, ultimately helping deaf and hard of hearing students achieve academic success. Based on this discussion, this study proposes Hypothesis 3: Rumination significantly predicts procrastination behavior in deaf and hard of hearing college students, with psychological resilience acting as a moderating factor.

### 2.4. The Present Study

**Figure 1:**
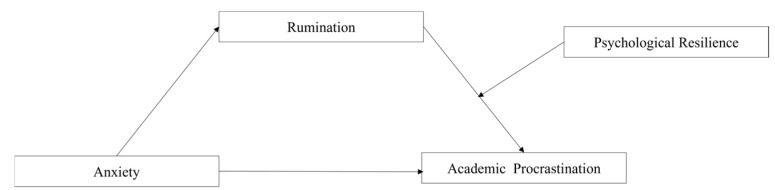
The proposed moderated mediation model.

This study aimed to explore the effects of anxiety and rumination on academic Procrastination in college students, and to analyze the mediating effect of rumination and the moderating effect of psychological resilience. Therefore, this study proposed a moderated mediation model (Figure 1)?

## 3. Method

### 3.1. Participants

It is generally accepted that a ratio of 10:1 between the number of subjects and the number of items would be most appropriate [49,50]. A total of 720 questionnaires were distributed to deaf and hard of hearing college students across three universities in Heilongjiang, Hunan, and Jiangsu provinces. After excluding incomplete or patterned responses, 685 valid questionnaires were obtained, yielding an effective response rate of 95.14%. The sample consisted of 328 females (47.9%) and 357 males (52.1%), with participants ranging in age from 17 to 25 years (M = 20.99, SD = 1.73), and 596 (87%) deaf and hard of hearing students from low-income families. Of all participants, 341 (49.8%) college students had congenital hearing loss. Regarding the degree of hearing loss, 553 (80.7%) were extremely severe (≥90 dB), 91 (13.3%) were severe (81–90 dB), 32 (4.7%) were moderate (61–80 dB), and 9 (1.3%) were mild (41–60 dB).

Prior to conducting the tests, we paid special attention to the reading comprehension levels of the deaf and hard of hearing college students. We proactively contacted their instructors to gather detailed information on their reading abilities. Based on this information, we are confident that the written test content was understandable to them. Additionally, we informed all participants beforehand that they were free to withdraw at any time if they encountered difficulties in understanding the test content. This measure was implemented to respect their rights and minimize any potential bias due to comprehension issues. Informed consent was obtained from both administrators and students before the survey was administered. Upon completion, each student received a small gift as a token of appreciation. The study was approved by the Ethics Committee of Nanjing Normal University of Special Education, ensuring adherence to ethical standards throughout the research process.

### 3.2. Instruments

#### 3.2.1. Generalized Anxiety Disorder Scale (GAD-7)

The Generalized Anxiety Disorder Scale (GAD-7) was employed to screen for generalized anxiety symptoms and assess the severity of anxiety over the past two weeks [51]. The scale comprises seven items, each rated on a 4-point Likert scale (0 = Not at all, 3 = Nearly every day), with higher scores indicating more severe anxiety. This scale has been validated for use among Chinese Deaf and Hard of hearing population [52]. In this study, the scale demonstrated strong internal consistency, with a Cronbach’s *α* coefficient of 0.94.

#### 3.2.2. Academic Procrastination Scale

The Academic Procrastination Scale (APS-S) was used to evaluate the extent to which procrastination affected students’ academic tasks [53]. The scale consists of five items, rated on a 5-point Likert scale ranging from 1 (Disagree) to 5 (Agree), with higher scores reflecting more severe academic procrastination among deaf and hard of hearing college students. This scale has been validated for use among Chinese college students [54]. The scale showed acceptable reliability in this study, with a Cronbach’s *α* coefficient of 0.81.

#### 3.2.3. Rumination Scale (RRS)

Developed by Nolen-Hoeksema and Morrow and later revised by Han and Yang, the Rumination Scale (RRS) assesses the tendency to engage in rumination, a form of repetitive negative thinking [55,56]. The scale includes 22 items divided into three dimensions: symptom rumination, compulsive thinking, and reflective pondering. Each item is rated on a 4-point Likert scale ranging from 1 (Never) to 4 (Always), with higher scores indicating a greater tendency toward rumination. This scale is well suited for Deaf and Hard of hearing College Students in China [36]. In this study, the scale’s reliability was supported by a Cronbach’s α coefficient of 0.97.

#### 3.2.4. Psychological Resilience Scale

The Psychological Resilience Scale, developed by Connor and Davidson and revised by Yu and Zhang, was utilized to measure the resilience of participants [42,57]. The scale comprises 25 items across three dimensions: tenacity, strength, and optimism. Each item is rated on a 5-point scale (1 = Never, 5 = Always), with higher total scores indicating greater psychological resilience. This scale has been widely used among the Chinese deaf and hard of hearing population, demonstrating high reliability [58,59]. In this study, the scale demonstrated adequate internal consistency, with a Cronbach’s *α* coefficient of 0.97.

### 3.3. Statistical Analysis

Data were processed and analyzed using SPSS 26.0 software along with the Process 3.3 macro. Initially, SPSS 26.0 was used for data entry, organization, descriptive statistical analysis, and correlation analysis. Subsequently, Hayes’ Process macro was applied for confidence interval estimation using the Bootstrap method, with 5000 resamples, to calculate a 95% confidence interval (CI) [60].

## 4. Results

### 4.1. Common Method Deviation

Data collected through self-reporting can introduce common method bias. To mitigate this, the study implemented procedural controls, including anonymous surveys and reverse scoring of some items. Additionally, Harman’s single-factor test was used to assess common method deviation [61]. An unrotated principal component factor analysis of all items revealed seven factors with eigenvalues greater than 1. The first common factor accounted for 31.11% of the cumulative variance, which is below the 40% threshold, indicating that common method bias is not a significant issue in this study.

### 4.2. Descriptive Statistics and Correlation Analysis of Variables

As shown in Table 1, anxiety, academic procrastination, and rumination among deaf and hard of hearing college students were positively correlated with each other, while psychological resilience was significantly negatively correlated with academic procrastination and rumination. Additionally, being from a low-income family was significantly correlated with anxiety, academic procrastination, and rumination. Therefore, this variable was included as a control in the subsequent model analysis.

### 4.3. Mediation Effect Test

To examine the mediating role of rumination between anxiety and academic procrastination in deaf and hard of hearing college students, Model 4 from Hayes’ PROCESS macro in SPSS was employed [60]. After controlling for the low-income family variable, the results are presented in Table 2. The first-step results indicated that anxiety significantly predicted rumination (*β* = 0.60, *p* < 0.001). The second step showed that anxiety significantly predicted academic procrastination (*β* = 0.29, *p* < 0.001). In the third step, rumination significantly predicted academic procrastination (*β* = 0.34, *p* < 0.001), and the positive prediction of anxiety on academic procrastination remained significant (*β* = 0.09, *p* < 0.001). These results suggest that rumination mediates the relationship between anxiety and academic procrastination in deaf and hard of hearing college students. The indirect effect was further tested using the bias-corrected percentile bootstrap method. The mediation effect was 0.20, *SE* = 0.03, 95% CI [0.14, 0.27], accounting for 68.75% of the total effect. Thus, rumination has a significant partial mediating effect between anxiety and academic procrastination.

### 4.4. Moderated Mediation Model Test

To explore the moderating role of psychological resilience between rumination and academic procrastination, Model 14 from Hayes’ PROCESS macro in SPSS was used to test the moderated mediation effect [60]. After controlling for the low-income family variable, the results are shown in Table 3. The interaction between rumination and psychological resilience significantly predicted academic procrastination (*β* = −0.06, *p* < 0.05). This finding indicates that psychological resilience significantly moderates the latter part of the mediation model between rumination and academic procrastination.

**Figure 2:**
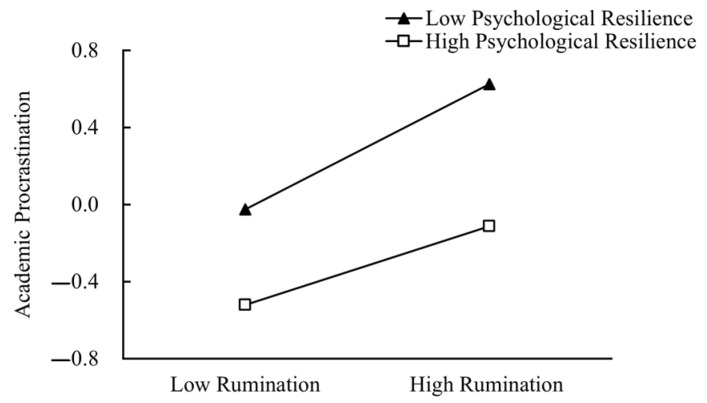
The moderating role of psychological resilience.

To further analyze this moderating effect, participants were divided into two groups based on their psychological resilience scores: low resilience (M − 1SD) and high resilience (M + 1SD). The predictive effect of rumination on academic procrastination was then examined separately for each group. As illustrated in Figure 2, among deaf and hard of hearing college students with low psychological resilience, rumination had a significant positive effect on academic procrastination (βsimple slope = 0.33, *p* < 0.001). For those with high psychological resilience, rumination also significantly predicted academic procrastination, but the effect was smaller (βsimple slope = 0.20, *p* < 0.001).

## 5. Discussion

In higher education, student mental health and academic performance are critical issues. In recent years, academic procrastination has become increasingly common among college students, negatively impacting their academic performance and mental health. This problem also exists for deaf and hard of hearing college students. The purpose of this study is to explore the mental health issues of deaf and hard of hearing college students and the impact of these issues on their academic performance and well-being. We view deaf and hard-of-hearing individuals not as a group requiring special care, but as individuals entitled to the same rights and opportunities as their hearing peers. By understanding the needs and experiences of this group, we can better understand the challenges they face in college and design more inclusive educational environments and support strategies to help them reach their full potential.

### 5.1. Anxiety and Academic Procrastination in Deaf and Hard of Hearing College Students

This study, focusing on deaf and hard of hearing college students as a unique group, demonstrated that anxiety significantly positively predicts academic procrastination, indicating that anxiety is a detrimental factor contributing to procrastination, thereby validating Hypothesis 1. This finding is consistent with previous research results [11,62]. According to attentional control theory, anxiety impairs an individual’s inhibitory control, which is a core component of the central executive system [39]. Specifically, anxiety impairs cognitive functions such as attention control and working memory, making it difficult for individuals to focus on academic tasks and manage anxiety-related intrusive thoughts. As a result, highly anxious individuals are more susceptible to distractions from irrelevant internal stimuli, allocating more cognitive resources to internal triggers, which leads to resource depletion and difficulty maintaining focus, thereby contributing to academic procrastination. Compared to hearing individuals, deaf and hard of hearing students do generally have a higher risk of developing various mental health problems [17]. However, for deaf and hard of hearing students, the cause of psychological symptoms such as anxiety cannot simply be attributed to deafness itself. Specifically, deafness is not the cause of these symptoms, and the root of the problem lies in inappropriate or incomplete medical care and education early in life, particularly the effects of early loss of access to visual language [63,64]. It should not be overlooked that the language deprivation of deaf and hard of hearing students is a social phenomenon. For a long time, it was believed that sign language would interfere with the oral language development of deaf and hard of hearing children, and sign language was consistently excluded as a primary or supplementary language intervention option for deaf and hard of hearing children [6]. Research suggests that the poor language performance of deaf and hard of hearing children may be due to a lack of visual language, such as sign language, during critical periods of language learning [8].

Some literature emphasizes the close relationship between language and behavior problems, and the term “language deprivation syndrome” has been coined to emphasize the range of symptoms that can result from language deprivation [65]. These associations warrant further research to guide interventions that target these deaf mental health issues. Early assessment of a deaf and hard of hearing child’s language skills is therefore crucial, especially primary prevention of visual language when deafness is recognized. For example, immersing deaf and hard of hearing children in a sign language-rich environment may reduce the risk of harm due to language deprivation. In addition, higher educational institutions need to address the learning needs of deaf and hard of hearing students by providing targeted and diversified support services. First, in the learning environment, the visual nature of deaf and hard of hearing students’ information reception should be considered, making information accessible through various visual means. For instance, using electronic screens for repeated display and leveraging technologies such as real-time speech recognition to convert spoken language into visual text can address communication barriers. Second, differentiated teaching support is essential, as deaf and hard of hearing students differ from hearing students in perception, attention, and thinking. Teachers must recognize that many deaf and hard of hearing students enter college with conceptual gaps and limited knowledge backgrounds, requiring more time to teach certain subjects. In inclusive classrooms, course content and methods should be continuously adjusted to provide various forms of scaffolding to meet the diverse needs of deaf and hard of hearing students and achieve educational goals. Lastly, academic and psychological counseling should be enhanced, and support such as sign language interpreters and peer note-takers should be arranged to help deaf and hard of hearing students adapt to inclusive education environments. When researching deaf populations, it is essential to acknowledge that power and status inequalities can affect research outcomes and influence how deaf and hard of hearing individuals are perceived by the hearing world. These biases often unfairly attribute challenges to deaf individuals themselves, reinforcing a flawed educational system based on the misconception that “Deaf can’t.”. In order to overcome this prejudice, some studies suggest actively involving deaf and hard of hearing people in the research process and establishing an equal partnership between deaf people and researchers [66]. This includes forming bilingual teams to ensure smooth communication between the two sides and to offset the potential bias that may be introduced by the hearing advantage. Therefore, in order to study the lifestyle of the deaf community in depth and avoid the negative impact of hearing center bias on deaf research, we should take the initiative to invite deaf and hard of hearing students to join the research team and participate in the whole research process.

### 5.2. The Mediating Role of Rumination

This study found that rumination significantly mediates the relationship between anxiety and academic procrastination in deaf and hard of hearing college students, supporting Hypothesis 2 and aligning with previous research [37]. Prior studies indicate that rumination in response to negative emotions is a relatively common and stable coping strategy. On the one hand, anxious individuals may ruminate as they attempt to evaluate their emotions and circumstances, hoping to find solutions or alleviate symptoms. However, research shows that rumination often leads to an excessive focus on negative emotions, with individuals dwelling on the causes and consequences of their anxiety. This prevents constructive action and creates a vicious cycle of negative emotions, rumination, and procrastination. Additionally, understanding rumination in deaf and hard of hearing students requires considering their environmental context and how interactions between individuals and their surroundings shape rumination patterns. Cognitive vulnerability theory suggests that early negative experiences can foster the development of negative self-schemas [67,68]. These schemas may cause individuals to adopt maladaptive coping strategies when faced with stressors in adulthood, resulting in negative emotional and behavioral outcomes. Deaf and hard of hearing students, who often experience discrimination, social exclusion, and communication barriers, may develop habitual negative coping patterns due to these cumulative risk factors, especially when exposed to negative early-life experiences. Rumination is also directly associated with academic procrastination [40]. The cognitive resource depletion model suggests that rumination drains cognitive resources, reducing the likelihood of individuals adopting positive coping strategies in response to negative events. Rumination can impair thinking, problem-solving, and goal-oriented behavior. Even when faced with effective solutions, individuals who ruminate excessively may lack the motivation to act on them, contributing to procrastination [69]. Consequently, rumination plays a critical role in shaping the mental health and academic success of deaf and hard of hearing college students. Psychological interventions targeting rumination in deaf and hard of hearing students should focus on reducing its negative effects. Techniques such as positive rumination training and cognitive–behavioral therapy can be employed to decrease compulsive thoughts and symptom-focused rumination, while fostering constructive reflection. Additionally, identifying and utilizing protective resources is crucial. Family, school, and community support systems can be leveraged during interventions to help reduce rumination. Educational institutions must also consider the unique needs of deaf and hard of hearing students, addressing systemic barriers that hinder communication and interaction. Developing more effective social support networks will promote greater engagement and well-being for deaf and hard of hearing students, enhancing their academic success and reducing procrastination.

### 5.3. The Moderating Role of Psychological Resilience

This study also found that psychological resilience significantly moderates the latter part of the mediation model, specifically the path “anxiety → rumination → academic procrastination.” In particular, rumination has a stronger impact on academic procrastination among deaf and hard of hearing college students with low psychological resilience, compared to those with high resilience, confirming Hypothesis 3. These findings suggest that psychological resilience can buffer the negative effects of rumination on academic procrastination. This conclusion aligns with the conditional model of psychological resilience, which posits that protective factors can moderate or mitigate the adverse effects of risk factors on developmental outcomes [70]. When individuals experience negative thinking, resilience as a personal trait helps them recover and adapt to difficulties, setbacks, and adversity [71] According to the psychological resilience framework, individuals with high resilience are better equipped to manage negative emotions in a constructive manner, enabling them to address problems more effectively [72]. Moreover, highly resilient individuals tend to focus on and process positive aspects of their experiences, which helps reduce the negative impact of adverse events, lowers the occurrence of irrational beliefs, and decreases the detrimental effects of rumination on academic procrastination [73,74]. Enhancing the psychological resilience of deaf and hard of hearing college students can be achieved by developing their coping mechanisms, emotional regulation skills, and problem-solving abilities. Research indicates that psychological resilience is fostered through interactions within a supportive environment [75,76]. This requires shifting attention to the environments in which deaf and hard of hearing individuals grow up—especially the obstacles and challenges present in these environments. We must explore how to integrate their resources with these challenges and consider how systems can further nurture resilience in young people. It is important to note that improving the resilience of deaf and hard of hearing students is not solely dependent on individual development and family support but also involves addressing broader social and structural barriers that contribute to risk and adversity. In conclusion, from an ecosystem perspective, personal resilience development requires not only strengthening internal resources and individual capacities but also fostering positive interactions between individuals and the broader systems in which they live. Access to communication technologies, stronger interpersonal connections, engagement within the deaf and hard of hearing community, and social skills training all contribute to positive outcomes for individuals with hearing loss. Schools can further support deaf and hard of hearing students by organizing structured, inclusive activities that encourage prosocial behavior and social participation. Such initiatives expose students to positive peer and adult role models, helping them build essential skills, a sense of belonging, and achievement, ultimately benefiting their overall development and reducing academic procrastination. In addition, deaf and hard of hearing people and experts in the field of hearing research emphasize the importance of deaf epistemology. Deaf epistemology refers to the unique body of knowledge constructed, shared, and transmitted by the deaf community about the world they inhabit [77]. It advocates respect for the cultural diversity, language practices, and lifestyles of the deaf community and their close connection to the educational system. At the heart of this epistemology is the belief that the quality of deaf education can only be effectively improved by fully recognizing the belief systems and worldviews of deaf and hard of hearing people. Research has shown that when educators, policy makers, and administrators are influenced by deaf epistemology, the academic performance of deaf and hard of hearing students improves significantly [78]. This finding further demonstrates the need to embrace deaf epistemology in order to improve the educational environment for deaf and hard of hearing people. Deaf epistemology not only helps us better understand the unique needs and characteristics of the deaf community, but also guides us on how to better meet those needs and improve the effectiveness of special education. In addition, educators need to be deeply aware of the unique potential and advantages of deaf and hard of hearing children in visual perception, visual learning, and visual language communication [79]. They can develop strong visual language skills independent of their hearing ability. Therefore, in building a deaf education system, we should make full use of these advantages and promote the growth and development of deaf students through visual learning. Accepting the deaf epistemology not only helps us to gain a deeper understanding of the deaf community from a different perspective, but also enables us to truly recognize the importance of visual learning, thereby building a more effective and appropriate education system for deaf and hard of hearing students.

### 5.4. Limitations and Future Directions

This study has some limitations that should be addressed in future research. First, the use of cross-sectional data in this study limits the ability to draw causal inferences. Future research should consider employing experimental designs or longitudinal approaches to better establish causality. Second, this study focused exclusively on psychological resilience as a moderating variable. Future studies should explore the potential moderating effects of other positive psychological traits, such as academic self-efficacy and optimism, on academic procrastination. Lastly, the reliance on self-reported data from deaf and hard of hearing college students may have introduced social desirability bias. To enhance the objectivity of the findings, future research could incorporate qualitative methods, such as in-depth interviews or observational studies.

## 6. Conclusions

This study yielded several important findings: (1) Anxiety is a positive predictor of academic procrastination among deaf and hard of hearing college students; (2) Rumination serves as a partial mediator in the relationship between anxiety and academic procrastination in this population; and (3) Psychological resilience moderates the mediation model, particularly in the path where anxiety influences academic procrastination through rumination. Notably, for deaf and hard of hearing students with low psychological resilience, rumination has a stronger impact on academic procrastination.

## Figures and Tables

**Table 1:** Descriptive statistics and correlations among variables (*n* = 685).

Variable	M	SD	1	2	3	4	5
1. LiF	1.13	0.34	1				
2. Anxiety	13.95	5.45	−0.10 **	1			
3. AP	14.97	3.83	−0.11 **	0.30 **	1		
4. Rumination	50.69	14.40	−0.10 **	0.60 **	0.40 **	1	
5. PR	70.98	18.74	−0.02	−0.02	−0.33 **	−0.15 **	1

Note: Low-income Families = LiF, Academic Procrastination = AP, Psychological Resilience = PR. M is mean value; SD is standard deviation. ** *p*  <  0.01.

**Table 2:** Mediation Effect Test (*n* = 685).

Dependent Variable	Independent Variable	*R* ^2^	*F*	*β*	*SE*	*t*
Rumination	LiF	0.36	194.88 ***	−0.04	0.09	−1.28
	Anxieties			0.60	0.03	19.47 ***
AP	LiF	0.10	36.46 ***	−0.08	0.11	−2.23*
	Anxieties			0.29	0.04	7.98 ***
AP	LiF	0.17	45.92 ***	−0.07	0.10	−1.94
	Rumination			0.34	0.04	7.66 ***
	Anxieties			0.09	0.04	2.08 *

Note: * *p* < 0.05, *** *p*  <  0.001.

**Table 3:** Moderated Mediation Model Test (*n* = 685).

Dependent Variable	Independent Variable	*R* ^2^	*F*	*β*	*SE*	*t*
AP	LiF	0.25	47.22 ***	−0.08	0.03	−2.46 *
	Rumination			0.26	0.04	6.28 ***
	PR			−0.31	0.03	−9.03 ***
	Rumination × PR			−0.06	0.03	−2.44 *

Note: * *p*  <  0.05, *** *p * <  0.001.

## Data Availability

The data presented in this study are available on request from the corresponding author. The data are not publicly available due to privacy or ethical restrictions.
